# The dying parent and dependent children: a nationwide survey of hospice and community palliative care support services

**DOI:** 10.1136/bmjspcare-2019-001947

**Published:** 2020-03-09

**Authors:** Jane Cockle-Hearne, Elizabeth Reed, Jennifer Todd, Emma Ream

**Affiliations:** 1 School of Health Sciences, University of Surrey, Guildford, UK; 2 Research Department, Princess Alice Hospice, Esher, UK

**Keywords:** hospices, palliative care, parental death, children, family, bereavement support

## Abstract

**Background:**

Annually, across the world a substantial number of dependent children experience the death of a parent through life-limiting illness. Without support, this has long-term implications for children’s emotional, social and physical well-being, impacting on health and social care services globally. Limited information exists on how service providers are meeting family needs when a parent with dependent children is dying.

**Aim:**

To determine the bereavement support provided to families with dependent children by UK hospices before and after a parent’s death.

**Design:**

A 23-item, cross-sectional, web-based survey of adult UK hospices. Closed and open-ended questions were asked about the features of support provided; open-ended response was sought to a question about the challenges faced by hospices in delivering support. Descriptive and non-parametric statistics and framework analysis were used to analyse the data.

**Results:**

197 hospices were invited to participate. Response rate was 66% (130/197). More types of support were provided after, than before, parental death (mean 6.36/5.64, z=−5.767, p<0001). Twenty-two per cent of hospices reported no formal processes for asking or documenting the presence of dependent children. Volunteers were an underused resource before parental death. Four themes characterised challenges in delivering support for families: emotional difficulties for families; practical and social difficulties for families; funding/resources; and staff training/numbers.

**Conclusions:**

Family needs are not consistently being met when a parent is dying. Areas for development include: enhanced systems to record when patients have dependent children; flexible approaches to support vulnerable families; staff training to help communication with families and management of their own fears of making the situation worse. Effective educational interventions and service developments to better support staff, parents and children are needed.

## Introduction

The death of a parent is highly traumatic for dependent children; without appropriate support it can have long-term effects on schooling, relationships, independence and emotional well-being.^1–5^ The number of children who experience the death of a parent is significant. In the USA, over 1.5 million children are living in single-parent households due to a parent’s death^6^; in Canada, it is estmated that 1 in 14 children will experience the death of a parent or sibling by the time they turn 18 7; in the UK in 2015, a total of 23 600 parents died, leaving an estimated 41 000 bereaved children.^8^

Losing a parent through prolonged illness can cause higher levels of maladaptive grief or post-traumatic stress for children than through sudden death; approximately half of children who lose a parent to cancer experience unresolved grief up to 9 years later.^4 9^ In the absence of suitable social support, transparent communication and cohesive family relationships, children can find grief, and their ability to adapt, difficult to manage.^2 10–13^ The psychological health of surviving parents can also deteriorate around bereavement, compounding parenting difficulties.^14^ However, if parents are guided to meet children’s needs, this can strengthen the family unit and build a protective environment as the family moves through the impending death and into bereavement.^15^ Both parents and children would like help from healthcare professionals in how to talk about a parent’s life-limiting illness.^16 17^

In 2014, the WHO called for countries to integrate palliative care support into their national health services. The UK palliative care service is acknowledged as one of the most comprehensive, along with services in Australia, Belgium, France, Germany, Ireland, the Netherlands, New Zealand, Singapore and Spain.^18^ However, there are still relatively few well-developed national palliative care strategies: continued evidence across countries indicates that bereavement support in palliative care services is typically generic and that the individual needs of affected families, especially children, are not being met.^18–20^

Hospices provide palliative and end-of-life care to people, either as inpatients or in the community, from the time they receive a life-limiting diagnosis to the end of their life. UK hospices care for over 200 000 people annually, accounting for 44% of all those likely to need expert end-of-life care.^21 22^ They offer multidisciplinary support encompassing clinical, physical, emotional, social and spiritual needs and provide bereavement support for some 49 000 people a year, as well as many more through wider networks of families and carers.^21 22^ Despite 5%–10% of hospice patients having children under 18 years,^21^ little is known about the support hospices provide for families prior to, or following, the death of a patient with dependent children. Greater knowledge of the nature of this specific hospice provision is important if the prebereavement and bereavement needs of this group of patients and their families are to be met.

To help achieve this we undertook a nationwide survey to understand the features of support provided in UK hospices to families with dependent children under 18 years, before and after parental death.

## Methods

We conducted a cross-sectional, web-based survey of UK hospices, defined as ‘organisations delivering adult in-patient and community palliative care services’ and ‘organisations delivering only community-based adult palliative care services’. The study was formally assessed through the authors’ University research governance procedures. The University’s online Self-Assessment for Ethics (SAFE) 2017 screening protocol was formally completed; the response received indicated that ethical committee review of the study was not required since no personal data were collected and there was low risk associated with participation^23^ (see online supplementary files 1 and 2). The University’s Code of Good Research Practice was followed throughout survey development, conduct and analysis.^24^ Reporting followed the Checklist for Reporting Results of Internet E-Surveys (CHERRIES) guidelines.^25^

10.1136/bmjspcare-2019-001947.supp1 Supplementary data

10.1136/bmjspcare-2019-001947.supp2 Supplementary data

### Survey design

The survey design was informed by literature synthesis and the authors’ expertise in hospice care. Topics incorporated are presented in box 1 (see online supplementary file 3 for questions asked). The survey was piloted in six hospices; no amendments were needed. It took 10–15 min to complete and was conducted through Qualtrics software,^26^ branded with the research team’s institutional logos. No password was necessary. Both closed and open-ended questions were used in an adaptive format with one question presented per screen page. One open-ended question asking about challenges experienced in supporting families with dependent children was asked of all participants who moved through the survey, irrespective of the questions they had already answered. Hospice size and region were asked.

10.1136/bmjspcare-2019-001947.supp3 Supplementary data

Box 1 **Survey topics**

**Types of support provided (before and after parental death)**

- Written information, CDs/DVDs, signposting to outside support services, signposting to web-based resources, one-to-one support sessions (face-to-face or telephone), groups or pair support sessions, peer group meetings, other.

**Who delivers support (before and after parental death)?**

- Doctor, nurse, social worker, psychologist/psychiatrist, specialist counsellor, chaplain, volunteer, other.

**Who receives support before parental death?**

- Patient individually, partner individually, child(ren) without either parent, the family, patient and partner together, partner and child(ren) together.

**Who receives support after parental death?**

- Partner individually, child(ren) without their surviving parent, partner and child(ren) together.

**Settings where support is delivered (before and after parental death)**

- Within the inpatient unit, in the patient’s family home, in community-run locations, over the internet, over the telephone, other.

**Evaluation of support provided**

- Carried out evaluation and willing/not willing to share.
- No evaluation.

**Practice for recording information about dependent children**

- Procedures for collecting information about patients’ dependent children.
- Support for staff to have conversations with patients about dependent children.

**Challenges experienced in supporting families with children under 18 years**

- Free text response.

**Hospice characteristics**

- Regional location.
- Number of beds.
- Number of new referrals per year.

### Sampling, recruitment and consent

Adult hospices in the UK were identified through a national directory compiled by Hospice UK. Email invitations were sent to the identified person responsible for providing patient support in each of the 197 adult member hospices. Recipients were asked to pass on the survey link to the appropriate member of their hospice staff if someone else was more qualified to participate. The invitation contained survey details and a secure survey link (online supplementary file 4). The survey landing page provided full participant information and asked for consent by informing participants that by starting the survey they would be agreeing to the use of the data in meeting the survey objectives (online supplementary file 5). The survey ran from 20 February to 12 April 2018. Two reminders were sent to all invitees; no incentives were offered for participation.

10.1136/bmjspcare-2019-001947.supp4 Supplementary data

10.1136/bmjspcare-2019-001947.supp5 Supplementary data

### Data analysis

Survey responses were analysed using Microsoft Excel (2013) and SPSS (V.24) software packages. Landing page-only visits were identified through log data and removed; partially complete surveys were included in the final analysis if at least the first question had been answered. Duplicate attempts were identified through log data; the most complete attempt retained. Descriptive statistics were used to analyse the survey data; percentages were based on the number of hospices answering each question. A Wilcoxon signed-rank test was used to evaluate the difference in the number of support modalities used by hospices before and after parental death. Open-ended responses were entered into QSR NVivo (V.11), coded and analysed for explanation of previous answers. Text responses describing challenges experienced by hospices in supporting families were coded and analysed with framework analysis.^27^

## Results

### Response rates

The survey landing page was visited 175 times, 130 individual hospices took part in the survey. The response rate was 66% (130/197); the full completion rate was 85% (111/130).

### Respondent characteristics

Hospices across all UK regions responded. Nearly 20% of responses were from London and the South East, the smallest response (≤4%) came from Wales and Northern Ireland. Slightly over half had ≤16 beds; nearly a quarter had under 500 referrals a year, and over half had between 501 and 2000 annual referrals. Community-based only palliative care services represented around 11% of the sample (table 1).

**Table 1:** Characteristics of hospices that took part in the survey

	Participants	
	n (%)	
Region		
London and South-East	22	19.6
North-West	15	13.4
North-East	12	10.7
East of England	11	9.8
South-West	11	9.8
Scotland	9	8.0
Yorkshire and Humberside	9	8.0
West Midlands	9	8.0
East Midlands	7	6.3
Wales	4	3.6
Northern Ireland	3	2.7
Total*	112	100
Beds		
1–10	27	24.1
11–16	38	33.9
17–30	31	27.7
31–40	3	2.7
More than 40	1	0.9
Community-based service only	12	10.7
Total*	112	100
Annual referrals		
Under 500	26	23.2
501–1000	42	37.5
1001–2000	28	25.0
2001–3000	7	6.3
3001–4000	7	6.3
4001–5000	1	0.9
More than 5000	1	0.9
Total*	112	100

*Base: all hospices that responded to the question.

### Support provided

Table 2 summarises the support delivered by participating hospices. All hospices provided some form of support before and after parental death, but there were variations across time points and across features of support.

**Table 2:** Types of support provided, staff roles involved in delivery and recipients of support

	Prebereavement n (%)		Bereavement n (%)	
Types of support provided*	n=130		n=118	
Written materials: books/leaflets	125	(96.2)	117	(99.2)
Signposting to outside agencies	123	(94.6)	117	(99.2)
One-to-one, face-to-face support	115	(88.5)	109	(92.4)
Signposting to web-based support	114	(87.7)	102	(86.4)
Groups or pair sessions	75	(57.7)	87	(73.7)
One-to-one telephone/internet	75	(57.7)	85	(72.0)
Peer group meetings	49	(37.7)	69	(58.5)
CDs/DVDs	23	(17.7)	30	(25.4)
Other	35	(26.9)	34	(28.8)
Staff roles involved in delivery*	n=123		n=117	
Specialist counsellors	90	(73.2)	94	(80.3)
Nurses	84	(68.3)	65	(55.5)
Social workers	81	(65.9)	66	(56.4)
Chaplains	69	(56.1)	54	(46.1)
Volunteers	57	(46.3)	72	(61.5)
Doctors	51	(41.5)	26	(22.2)
Psychologists/psychiatrists	22	(17.9)	22	(18.8)
Other	22	(17.9)	22	(18.8)
Recipients of support*	n=119		n=115	
Patients individually	111	(93.3)	–	–
Partners individually	111	(93.3)	112	(97.4)
Patient and partner	106	(89.1)	–	–
Families together	100	(84.0)	–	–
Partner and child/children	97	(81.5)	95	(82.6)
Child/children without parent	90	(75.6)	89	(77.4)
Any type of support for children	116	(97.5)	99	(86.1)
No support for children	3	(2.5)	16	(13.9)

*Base: all hospices that responded to the question.

#### Types of support provided

Provision of written materials was most often reported before (96.2%) and after (99.2%) the death of a parent. Signposting to outside agencies was also highly reported (94.6% before; 99.2% after). The next most common types of support were individual face-to-face sessions (88.5%; 92.4%) and signposting to web-based support (87.7%; 86.4%). Group sessions, telephone/internet support, peer group meetings and CDs/DVDs were less commonly provided at either stage. Support described under 'other', before and after parental death, tended to be social support (trips. events) or creative activities (music, art). Remembrance, memory and legacy events were also offered after parental death.

**Figure 1:**
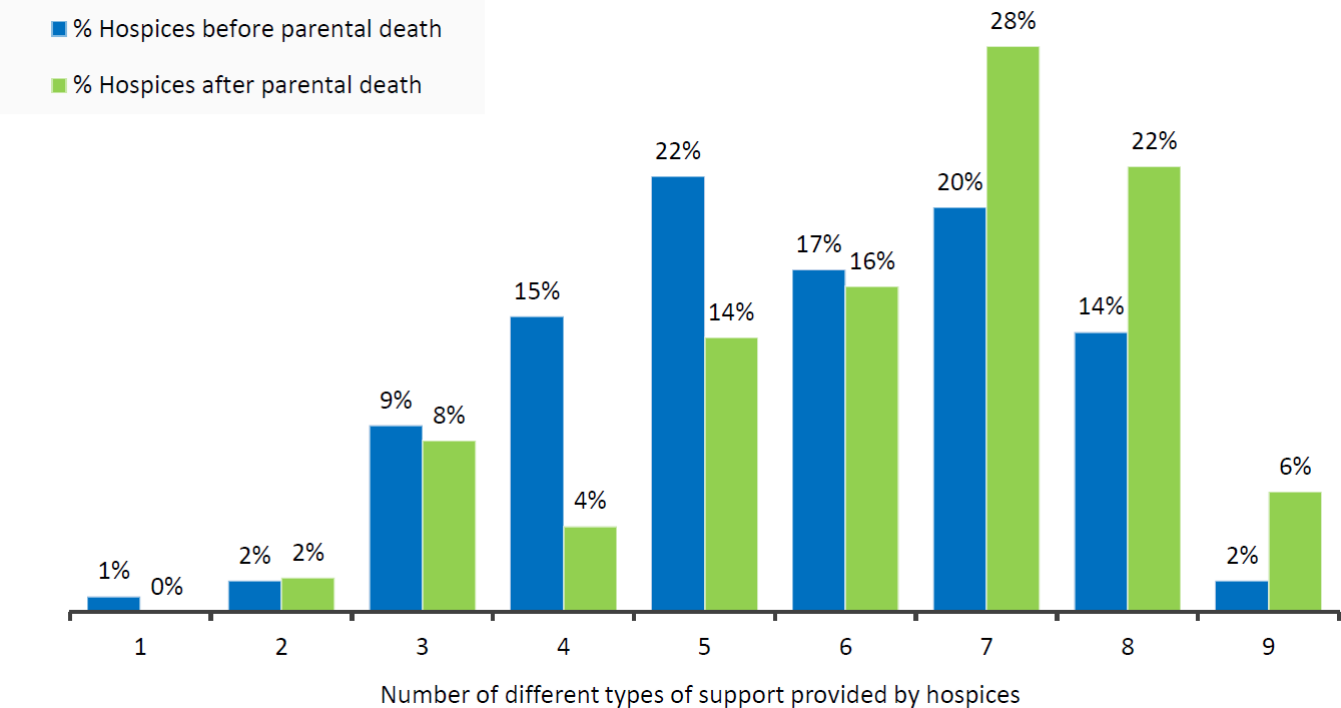
The number of different types of support provided by hospices for families with dependent children before and after parental death. *Percentages rounded, totals may be greater than 100%.*

Types of support provided across the sample were significantly higher in number (z=−5.767, p<0001 (two tailed) after parental death (mean 6.36, SD 1.712) than prior to it (mean 5.64, SD 1.689)) (figure 1).

#### Who delivers support?

Specialist counsellors were most commonly reported as being responsible for delivering support to families before and after parental death (73.2% and 80.3%, respectively). Nurses, social workers and chaplains also regularly delivered support but more likely before than after death. There were fewer reports of doctors delivering support, but twice as many did so before parental death than after. In contrast, more hospices involved volunteers after (61.5%) than before (46.3%) parental loss. Volunteers were involved in all types of support; they mostly delivered written information and signposting to outside agencies at any time point, but they were more likely to be involved in one-to-one or group sessions after parental loss (online supplementary file 6). The least reported professionals delivering support to families were psychologists and psychiatrists: only 17.9% of hospices reported their involvement before, and 18.6% after, parental death (table 2).

10.1136/bmjspcare-2019-001947.supp6 Supplementary data

#### Who receives support?

Before parental death most hospices reported supporting patients (93.3%) and partners (93.3%) individually; nearly as many reported supporting patients and partners together (89.1%) and families together (84.0%). After parental death, almost all hospices supported bereaved partners individually (97.4%).

Over 80% of hospices supported partners and their children together before the death of a parent; similarly, 80% did so after parental death, although these were not necessarily the same hospices. Children were supported on their own in three quarters of hospices before (75.6%) and after (77.4%) parental death. No support was provided to children in 2.5% of hospices before parental death and 13.9% of hospices after parental death (table 2).

#### Settings where support is delivered

Setting where support was delivered to families was similar before and after parental death for both inpatient and community-based hospices. Almost all inpatient hospices delivered support within their units but also offered telephone support and visits to the family home. A few community-based organisations indicated they delivered support in a hospice unit, but their main settings for providing support were in the family home or over the telephone. The internet was used by only a minority (table 3).

**Table 3:** Settings where support is delivered

	Inpatient hospices				Community-based hospices			
	Prebereavement		Bereavement		Prebereavement		Bereavement	
	n (%)		n (%)		n (%)		n (%)	
Hospices*	100		100		12		12	
Inpatient unit^†^	99	(99.0)	91	(91.0)	1	(8.3)	2	(16.7)
Over the telephone	78	(78.0)	76	(76.0)	7	(58.3)	7	(58.3)
Patient’s family home	68	(68.0)	69	(69.0)	9	(75.0)	7	(58.3)
Community-run locations	40	(40.0)	49	(49.0)	4	(33.3)	5	(41.7)
Over the internet	9	(9.0)	17	(17.0)	1	(8.3)	2	(16.7)
Other	43	(42.2)	49	(49.0)	9	(75.0)	8	(66.7)

* Base: All hospices that responded to the question.

†*Inpatient unit* defined as any premises on the hospice's own or other hospice site.

Details of additional settings were provided by 25 (21%) hospices for support before parental death, and by 19 (16.5%) hospices for support after death. Dependent children’s schools or colleges were a prominent setting. One hospice in a rural setting offered counselling via Skype both before and after parental death.

#### Evaluation of support provided

Nearly two-thirds of hospices reported carrying out evaluation or assessment of the support they provided (72/114, 63.2%). Twenty-one (18.4%) indicated that they would be willing to share their findings with the research team, but no hospices followed this through despite information for contacting the research team being provided in the survey.

### Practice for recording information about dependent children

Three-quarters (78.4%, 87/111) of hospices reported that they ask formally and record if patients have dependent children under 18 years. The remainder of hospices that responded (21.6%, 24/111) had no formal or systematic process for asking for, or recording, this information. Of the hospices that did ask and record, 78.2% (68/87) also formally supported their staff to undertake this work. The remainder, 21.8% (19/87), offered no formal support or training for staff in having conversations with patients about their children’s needs. Half of the responding hospices that provided support to staff outlined that this principally comprised generic and ad hoc preparation; little targeted support was provided. There was little mention of teamwork or team processes.

### Challenges experienced in supporting families with dependent children

We asked hospices about the challenges faced in delivering support to families with dependent children. Response was received from 98 hospices (87.5%). Verbatim comments are presented in box 2. Four overarching themes emerged.

Box 2 **Challenges faced in providing support services to families**

**Emotional difficulties for families**

‘*If the parent who is ill is struggling, they can insist that children are not told, which needs to be respected.*’

‘*Sometimes families may be reluctant to engage due to anxieties* [about] *upsetting children*. […] *we are asked not to mention the diagnosis when engaging with children.*’

‘*The challenge can often be encouraging families to have difficult discussions with children whose relative is poorly* [and at the] *end stages of life.*’

‘*Some parents want to appear strong and therefore the model of grief they present to their children informs the child that they must be strong also, this can have implications for how effective a service is as the child may not be as engaged as if they believed there would be a true benefit.*’

**Practical and social difficulties for families**

‘*When we have to rely on parents to bring their child to the hospice for their 1-1 sessions, this can be hit and miss therefore no consistency to the support the child receives.*’

‘*Sometimes the team are faced with families presenting with highly complex needs often late in their prognosis*. [For example] *lone parents who have children in foster care or who are living with other family members. This makes it more difficult* […] *meeting the* […] *needs of these families in the moment* […].’

‘*One of the main challenges in providing support to young families can be the chaotic lifestyle that some families from socially deprived areas experience. This can inhibit the surviving parent's ability to commit to regular attendance at the service.*’

‘*Another challenge is in encouraging adolescents to commit to and attend their scheduled sessions. This group tends to dip in and out of services.*’

**Funding/resources**

‘*In a world of increasing demand to measure/quantify/justify in order to attract funding, this ethos* [trust, openness and being-there, rather than “doing”] *there is a real challenge for the team to hold on to what is really important and not be distracted.*’

‘*The challenges we face are in procuring funding for counsellors and specialists in supporting families and children both pre and post bereavement.*’

‘*This service ceased due to funding issues. Many patients/partners are unable to travel to the Hospice due to serious illness and the demands of caring, so this has impacted on the number of people who can now avail of this service as out-patients.*’

‘*Often children have missed a lot of school time during the illness period of their parent, so the school and the parents want them to have out of hour’s appointments in early evenings, straight after school or on the weekend. We don't have the coverage of staff/volunteers to meet the full need.*’

**Staff training/numbers**

‘[…] *nurses find dealing with the emotional labour of caring for young patients and their dependent children particularly challenging because their training and background tends to be more about “doing” than “being”.*’

‘*Our challenges are about finding the right/qualified staff to deliver the care/support and to train others.*’

‘*Volunteer recruitment is currently providing our greatest limitation to providing support. Either getting volunteers in the first place or retaining for this specific client group.*’

#### Emotional difficulties for families

Hospices described a reluctance of some parents to discuss parental illness with their children in order to protect them from distress. Staff could find this challenging but respected parents’ wishes. When parents did not want to acknowledge the inevitability of death, the route for supporting children became *blocked* and children became *invisible*. Some parents presented a *strong*, independent role model that children followed, which hampered their engagement and the effectiveness of services. Opportunity and time to build trusting relationships with families was perceived important for supporting them in preparing children for parental death. If no support was provided before the death, provision afterwards was considered more challenging.

#### Practical and social difficulties for families

The time and cost involved for families in remote locations to access hospice support were barriers to engagement. Barriers were further identified in respect of social deprivation, cultural demands and spiritual beliefs. Some hospices explained how *chaotic* households could also hamper partners' and children’s engagement with services. The wide range of child development stages also presented challenges: children under 5 years, which is a time of speedy development, were often not provided for; teenagers could be erratic and avoid *commitment* to support. There was mention of inadequate electronic systems to record the presence of children in enough detail. A recurring challenge was the need to deliver services for children outside of education hours which impacted on staff time and availability.

#### Funding and resources

Many hospices lamented the continual need to secure funding to train and maintain staff for bereavement support services; they feared this took precedence over making time to provide *what is really important*. Withdrawal of funding had curtailed some support services, particularly in the community, leaving groups of people with no access to bereavement care. Many hospices could not meet the increasing demand caused by the longer waiting lists for external support organisations.

#### Staff needs

Significant *emotional labour* was involved in caring for young patients and their dependent children, yet nurses and social workers were not necessarily formally trained in family communication skills in end-of-life care; their training and background were described as *more about ‘doing’ than ‘being’*. Unskilled or inexperienced staff could be reluctant to engage with children for fear of *making matters worse*. Recruiting and retaining volunteers, on which many services relied heavily, was also a challenge for some hospices.

## Discussion

### Main findings

This is the first national survey of the support provided in UK adult hospices to families with dependent children when a parent is dying. We found that hospices clearly recognise and acknowledge the importance of helping parents to communicate with their children about what is happening at this time. Beyond this, we found that a greater proportion of hospices provide a range of family support after a parent’s death than before parental death, and that a substantial minority do not have any formal processes for recording family circumstances. The data also showed that a greater number of adult hospices provide support at their units compared with in the community or remotely. This does not reflect provision of palliative care generally of which 83% is provided in community-based settings.^21^ The survey had a good response rate, reflective of previous hospice survey work, indicating that participating services considered the topic important.^28 29^ This could have been encouraged by increasing social and media discourse related to bereaved young families. It could also have been positively affected by perceived endorsement of a well-known and respected hospice which generated the email invitations to the study. We believe that the views of hospice and community palliative services across the UK were captured well and provided representative insights into how this sector is meeting the needs of children and families when a parent is dying.

### Support before and after parental death

Several factors could contribute to the broader range of support being delivered to families following parental death than prior to it. Many people live for years with life-limiting illness before experiencing sudden decline in health, yet time between referral to palliative care and death can be very short.^30^ Prognostication is inherently difficult and managing patients’ often complex conditions may dominate over the needs of the wider family.^31–33^ Rapidity of decline may provide limited opportunity to assess and address children’s needs at this time, or to build relationships with parents to facilitate discussions about children’s needs. Limited time to support the needs of the wider family before parental death may also deter provision of shared or peer support: we found that social and remote forms of support were less common before death. Shared support requires planning and commitment, but before death more spontaneous and unstructured forms of support may be easier to deliver.

Some parents may not wish their children to be informed about impending death.^34^ Indeed, a *chain of protection* appeared evident from the survey. Staff tended to shy away from the difficult subject of parental death to protect parents from distress; parents themselves opted not to discuss it with their children to likewise protect them. Finally, as was clear here and in previous research, staff may feel unprepared to discuss with parents how best to prepare their children for parental death due to insufficient related training.^35^ This training gap clearly needs to be addressed to enhance staff skills, competence and confidence in addressing parents and children’s need for support around the time of parental loss.

### Support for children

It is clear from these data that hospices believe in the importance of relational care; support for both partners and patients was widespread across time points. However, support for children was less common, in particular, in the absence of other family members. This may be linked to our finding that a quarter of services across the UK do not have formalised processes for determining and recording patients’ family structures. What is more, among those hospices that do have formal processes, a sizeable minority do not provide staff with any kind of support to engage with families’ bereavement needs either before or after parental death.

Such variability risks some families’ needs being unassessed, with the result that children become invisible to the service. This shortcoming could be easily addressed. Sharing family details across team members through patient records is important and would enable a comprehensive team approach to meeting family needs. It is interesting to note that little reference was made spontaneously to multidisciplinary teamwork in managing children’s needs.

### Role of volunteers

The data from this survey revealed that volunteers, together with specialist counsellors, were most commonly involved in delivering support after parental death, yet they appear to be underused before parental death. There may be pragmatic reasons for this; since healthcare professionals have continuous contact with patients before death, this may preclude the need for volunteer involvement. Nevertheless, our data indicate that there is a gap to fill in supporting patients with families before the family moves into bereavement. A previous national survey of volunteering in UK hospices found 40% of hospices were working with people who volunteered mental health skills^29^; arguably this is a resource that could be developed. The size of the hospice volunteer sector and the time individuals provide are relatively stable over time,^36^ and the challenges of recruiting and retaining volunteers indicated in this survey may not be universal. Designing recruitment processes and responsibilities to meet volunteers’ motivational needs may be beneficial where such challenges are encountered. Volunteers are involved in hospice and palliative care services across a wide international framework and volunteering, historically essential in the development of the hospice sector, will continue to be a mainstay for its future.^36^

### Implications

UK national end-of-life guidance for adults repeatedly acknowledges that families should have honest, sensitive and well-informed conversations about dying, death and bereavement. Specifically, guidance states that dependent children require tailored support^37 38^ and, if patients with children want support, healthcare professionals should offer information and encourage family communication.^39^ To meet guideline requirements, this survey suggests that support for children and families within service models needs to be more proactive; hospices appear more attuned to addressing needs of children once they arise, rather than preventing them from arising.

Although this was a survey of UK hospices it has relevance in an international context. The hospice movement is developing globally and shared understanding of support delivery in the context of families with dependent children can help meet emerging and growing service requirements across different countries. It can also help determine research priorities to maximise provision.

### Limitations

The survey as intended provided a snapshot in time of the breadth and scope of support from an organisational perspective. We did not gauge the focus, quality and content of support provision, nor how much specific support families receive: this should be the subject of further exploratory work. We did, however, ask hospices if they would share evaluations of their intervention work with us but despite some expressed willingness, this did not happen. More incentives and further communication with hospices would be required to secure sharing of data.

While we believe we achieved a good representation of the breadth of UK hospices, we did not include hospital palliative care services, so cannot generalise our findings across the entire palliative care sector. Although we included open-ended questions in the survey, it is unlikely we were able to entirely capture the complexity of staff’s challenges in this sensitive area of work. Future work should concentrate on developing understanding of factors affecting how well staff across the palliative care sector engage with parents about the needs of their children and develop effective interventions to support staff and help them manage their own feelings of fear and diffidence.

## Conclusions

This UK survey has highlighted the need to enhance several features of service provision to ensure universal support is provided for families when a parent is dying, and to place practices more in line with national palliative care guidelines. Recording and assessing patients’ family circumstances and the presence of dependent children remains a key requirement to identify and flag vulnerable families. Staff require skills training and supervision to help them interact with families and manage their own fears of doing harm. Moreover, there are requirements for flexible services to enhance family commitment to support; expanded types of support before parental death; greater provision for children before and after parental death; greater community-based support; and greater involvement of voluntary support. This will provide foundations for families and their children that may prevent later problems.

## Data Availability

Data will be made available for verification purposes upon request as participants did not consent to data sharing beyond the research team. Details can be found at DOI: 10.5281/zenodo.3571335

## References

[1] Aynsley-Green A , Penny A , Richardson S . Bereavement in childhood: risks, consequences and responses. BMJ Support Palliat Care 2012;2:2–4. 10.1136/bmjspcare-2011-000029 24653486

[2] Ellis J , Dowrick C , Lloyd-Williams M . The long-term impact of early parental death: lessons from a narrative study. J R Soc Med 2013;106:57–67. 10.1177/0141076812472623 23392851 PMC3569022

[3] Huizinga GA , Visser A , Zelders-Steyn YE , et al . Psychological impact of having a parent with cancer. Eur J Cancer 2011;47:S239–46. 10.1016/S0959-8049(11)70170-8 21943981

[4] Kaplow JB , Howell KH , Layne CM . Do circumstances of the death matter? Identifying socioenvironmental risks for grief-related psychopathology in bereaved youth. J Trauma Stress 2014;27:42–9. 10.1002/jts.21877 24478197

[5] Tafà M , Cerniglia L , Cimino S , et al . Predictive values of early parental loss and psychopathological risk for physical problems in early adolescents. Front Psychol 2018;9. 10.3389/fpsyg.2018.00922 PMC5998644 29928249

[6] Owens D . Recognizing the needs of bereaved children in palliative care. J Hosp Palliat Nurs 2008;10:14–16.

[7] Child and Youth Grief Network Source: Statistics Canada, 2016 Census of Canada and 2016 Mortality. Available: https://www.childrenandyouthgriefnetwork.com/https://www12.statcan.gc.ca/census-recensement/2016 [Accessed 10 Dec 2019].

[8] Childhood Bereavement Network . Key Statistsics, 2015. Available: http://www.childhoodbereavementnetwork.org.uk/research/key-statistics.aspx [Accessed 31 Jan 2019].

[9] Bylund-Grenklo T , Fürst CJ , Nyberg T , et al . Unresolved grief and its consequences. A nationwide follow-up of teenage loss of a parent to cancer 6-9 years earlier. Support Care Cancer 2016;24:3095–103. 10.1007/s00520-016-3118-1 26899858

[10] Akerman R , Stratham J . Bereavement in childhood: the impact on psychological and educational outcomes and the effectiveness of support services. London: Child Wellbeing Research Centre and the Institute of Education, 2014.

[11] Christ GH , Christ AE . Current approaches to helping children cope with a parent's terminal illness. CA Cancer J Clin 2006;56:197–212. 10.3322/canjclin.56.4.197 16870996

[12] Luecken LJ , Roubinov DS . Pathways to lifespan health following childhood parental death. Soc Personal Psychol Compass 2012;6:243–57. 10.1111/j.1751-9004.2011.00422.x 23555319 PMC3613285

[13] Fearnley R , Boland JW . Parental life-limiting illness: what do we tell the children? Health Care 2019;7:47. 10.3390/healthcare7010047 PMC6473248 30897857

[14] Siegel K , Karus D , Raveis VH . Adjustment of children facing the death of a parent due to cancer. J Am Acad Child Adolesc Psychiatry 1996;35:442–50. 10.1097/00004583-199604000-00010 8919706

[15] MacPherson C . Telling children their ill parent is dying: a study of the factors influencing the well parent. Mortality 2005;10:113–26. 10.1080/13576270500102872

[16] Fearnley R , Boland JW . Communication and support from health-care professionals to families, with dependent children, following the diagnosis of parental life-limiting illness: a systematic review. Palliat Med 2017;31:212–22. 10.1177/0269216316655736 27383635 PMC5347362

[17] Bylund-Grenklo T , Kreicbergs U , Uggla C , et al . Teenagers want to be told when a parent's death is near: a nationwide study of cancer-bereaved youths' opinions and experiences. Acta Oncol 2015;54:944–50. 10.3109/0284186X.2014.978891 25467964

[18] Morrison RS . A national palliative care strategy for Canada. J Palliat Med 2018;21:S-63–75. 10.1089/jpm.2017.0431 29283876 PMC5733738

[19] Aoun SM , Rumbold B , Howting D , et al . Bereavement support for family caregivers: the gap between guidelines and practice in palliative care. PLoS One 2017;12:e0184750. 10.1371/journal.pone.0184750 28977013 PMC5627900

[20] Griese B , Burns MR , Farro SA , et al . Comprehensive grief care for children and families: policy and practice implications. Am J Orthopsychiatry 2017;87:540–8. 10.1037/ort0000265 28945443

[21] Hospice UK . Facts and figures. Available: https://wwwhospiceukorg/about-hospice-care/media-centre/facts-and-figures [Accessed 03 Sep 2019].

[22] Hospice UK . Hospice care in the UK 2016: scope, scale and opportunities. Available: https://wwwhospiceukorg/docs/default-source/What-We-Offer/publications-documents-and-files/hospice-care-in-the-uk-2016pdf?sfvrsn=0 [Accessed 03 Sep 2019].

[23] University of Surrey . Ethics Handbook for teaching and research, 2018. Available: https://www.surrey.ac.uk/sites/default/files/2018-12/ethics-handbook-version-3.3.pdf [Accessed 16 May 2019].

[24] University of Surrey . Code on good research practice, 2017. Available: https://www.surrey.ac.uk/sites/default/files/Code%20on%20Good%20Research%20Practice.pdf [Accessed 16 May 2019].

[25] Eysenbach G . Improving the quality of web surveys: the checklist for reporting results of Internet E-Surveys (cherries). J Med Internet Res 2004;6:e34. 10.2196/jmir.6.3.e34 15471760 PMC1550605

[26] Qualtrics . Provo, Utah, USA, First Released 2005, Copyright 2019. Available: https://www.qualtrics.com [Accessed Feb/April 2018].

[27] Ritchie J , Lewis J , McNauthton Nicholls C , et al . Qualitative research practice: a guide for social science students and researchers. London: SAGE, 2014.

[28] Nutley SM , Walter I , Davies HTO . Using evidence: how research can inform public services. Bristol: The Policy Press, 2007.

[29] Burbeck R , Low J , Sampson EL , et al . Volunteers in specialist palliative care: a survey of adult services in the United Kingdom. J Palliat Med 2014;17:568–74. 10.1089/jpm.2013.0157 24475743 PMC4012622

[30] Currow DC , Vergo MT , Ekstrom M . Sudden death in palliative care. J Pain Symptom Manage 2015;50:e1–2. 10.1016/j.jpainsymman.2015.06.009 26166182

[31] Carduff E , Johnston S , Winstanley C , et al . What does ‘complex’ mean in palliative care? Triangulating qualitative findings from 3 settings. BMC Palliat Care 2018;17:12. 10.1186/s12904-017-0259-z 29301524 PMC5753489

[33] Glare P , Virik K , Jones M , et al . A systematic review of physicians' survival predictions in terminally ill cancer patients. BMJ 2003;327. 10.1136/bmj.327.7408.195 PMC166124 12881260

[33] Gwilliam B , Keeley V , Todd C , et al . Development of prognosis in palliative care study (PIPs) predictor models to improve prognostication in advanced cancer: prospective cohort study. BMJ 2011;343:d4920. 10.1136/bmj.d4920 21868477 PMC3162041

[34] Aamotsmo T , Bugge KE . Balance artistry: the healthy parent's role in the family when the other parent is in the palliative phase of cancer--challenges and coping in parenting young children. Palliat Support Care 2014;12:317–29. 10.1017/S1478951513000953 24103392

[35] Franklin P , Arber A , Reed L , et al . Health and social care professionals' experiences of supporting parents and their dependent children during, and following, the death of a parent: a qualitative review and thematic synthesis. Palliat Med 2019;33:49–65. 10.1177/0269216318803494 30371147

[36] Scott R , Howlett S . The changing face of Volunteering in hospice and palliative care. Oxford University Press, 2018.

[37] National Institute of Health and Care Excellence . End of life care for adults, 2011. Available: https://wwwniceorguk/guidance/qs13/resources/end-of-life-care-for-adults-pdf-2098483631557

[38] National Palliataive and End of Life Care Partnership . Ambitions for palliative and end of life care: a national framework for local action 2015-2020. Available: http://endoflifecareambitionsorguk/wp-content/uploads/2015/09/Ambitions-for-Palliative-and-End-of-Life-Carepdf

[39] National Institute for Health and Care Excellence . Improving supportive and palliative care for adults with cancer, 2004. Available: https://wwwniceorguk/guidance/csg4/resources/improving-supportive-and-palliative-care-for-adults-with-cancer-pdf-773375005

