# Supplementary material for: The dying parent and dependent children: a nationwide survey of hospice and community palliative care support services
Source: BMJ Support Palliat Care. 2020 Mar 9;12(e5):e696–704. doi: 10.1136/bmjspcare-2019-001947 (PMC9606526; doi:10.1136/bmjspcare-2019-001947)
Supplement: Supplementary data [file bmjspcare-2019-001947supp001.pdf]

The dying parent and dependent children. A nation nationwide survey of hospice and community palliative care support provision.

Supplementary Fil. 1.

Self-Assessment Form: Ethics (SAFE)

100% complete

Thank you

Completion receipt

Receipt number: 160708-160702-26305270
Submission 2017-10-26 14:48:55

Thank you for completing the Self-Assessment Form: Ethics.

According to the answers you have provided your study does not meet the criteria for ethical review, and a submission to the University Ethics Committee is not required.

Note: Your *responses* should be downloaded and kept with your study documentation.

If you have any questions please feel free to contact us via [redacted]
