# Supplementary material for: The dying parent and dependent children: a nationwide survey of hospice and community palliative care support services
Source: BMJ Support Palliat Care. 2020 Mar 9;12(e5):e696–704. doi: 10.1136/bmjspcare-2019-001947 (PMC9606526; doi:10.1136/bmjspcare-2019-001947)
Supplement: Supplementary data [file bmjspcare-2019-001947supp002.pdf]

The dying parent and dependent children: a nationwide survey of hospice and community palliative care support services.

Supplementary File 2.

Self-Assessment Form: Ethics (SAFE)

Response ID		Completion date
160708-160702-26305270		26 Oct 2017, 14:48 (BST)
1	**Project title**	Supporting families preparing children for parental death: A survey among UK hospices.
2	**Chief Investigator:**	Professor Emma Ream.
2.a	**Email address:**	[redacted]
3	**Level of research**	Staff
3.a	**Co-investigators:**	Professor Faith Gibson (Surrey);<br>Dr Anne Arber (Surrey);<br>Jane Cockle-Hearne (Surrey);<br>Dr Liz Reed (Princess Alice Hospice);<br>Dr Jennifer Todd (Princess Alice Hospice).
4	**Does the study require review by an NHS Research Ethics Committee?**	No

Cockle-Hearne J, *et al. BMJ Support Palliat Care* 2022; 12:e696–e704. doi: 10.1136/bmjspcare-2019-001947

5	**Does the study involve the inducement of MORE than minimal stress to the participant?**	No
6	**Does the study involve children under 16 years or other vulnerable groups such as those 16 and over who may feel under pressure to take part due to their connection with the researcher?**	No
7	**Does the study involve prisoners or young offenders?**	No
8	**Does the study involve the new collection or donation of human tissue, as defined by the Human Tissue Act, from a living person or the recently deceased according to the Human Tissue Authority?**	No
9	**Does the study involve any of the following ...**	No

10	**Are you planning to access records of and/or collect personal confidential data, concerning identifiable individuals as defined by the UK Data Protection Act 1998?**	No
11	**Are you linking or sharing personal data or confidential information beyond the initial consent given (including linked data gathered outside of the UK)?**	No
12	**Will you collect or access audio/video recordings, photographs or quotations within which participants may be identifiable and with the intention to disseminate those beyond the research team?**	No

Cockle-Hearne J, *et al. BMJ Support Palliat Care* 2022; 12:e696–e704. doi: 10.1136/bmjspcare-2019-001947

**13**	**Does the study require participants to take part in the study without their knowledge and/or consent at thetime?**	No

**14**	**Does the study involve deceptionother than withholding information about the aims of the research until the debriefing?**	No
**15**	**Do you plan to offer incentives which may unduly influence participants' decision to participate?**	No
**16**	**Does the study involve activities where the safety/wellbeing of the researcher may be in question?**	No
**17**	**Do you think that any other significant ethical concerns may arise, or does your external funding body or sponsor require ethical review to be undertaken?**	No

**18**	**Could the behavioural/physiological intervention possibly lead to discovery of ill health or concerns about wellbeing in a participant incidentally even if the intervention in itself causes no more than minimal stress is to the research participant?**	No
**19**	**Are you investigating existing working or professional practices among participants, identifiable to yourself as the researcher atyour own place of work (this may be the University of Surreyor another organisation where you, your supervisor or coinvestigator work)?**	No
**20**	**Is the research proposal to be carried out by persons unconnected with the University, but wishing to use staff and/or students as participants?**	No

3 / 4

**21**	**I, the undersigned, confirm that I have read the Ethics Handbook for Teaching and Research and the Code on Good Research Practice. I understand that the project may be monitored and audited by the University of Surrey to ensure that it is carried out in accordance with good practice, legal and ethical requirements and any other guidelines. I understand that the protocol and any associated documents such as information sheets and consent forms should have version numbers and dates. If I make any significant changes to my protocol I understand that I should complete the self-assessment again. I am also aware that any knowingly wrong answers to any of the questions below and any research misconduct reported may lead to disciplinary measures after investigation. In case of dissertation projects or theses the provision of knowingly incorrect information or proven research misconduct may affect academic progression.**	I agree
**21.a**	**Name**	Jane Cockle-Hearne
**21.b**	**Date self-assessment form is submitted**	26/10/2017
