# Supplementary material for: The dying parent and dependent children: a nationwide survey of hospice and community palliative care support services
Source: BMJ Support Palliat Care. 2020 Mar 9;12(e5):e696–704. doi: 10.1136/bmjspcare-2019-001947 (PMC9606526; doi:10.1136/bmjspcare-2019-001947)
Supplement: Supplementary data [file bmjspcare-2019-001947supp003.pdf]

The dying parent and dependent children: a nationwide survey of hospice and community palliative care support services.

Supplementary File 3.

Survey Questions

Section 1.

In this first section, we would like to know about the support your organisation provides.

Q1. First, thinking about the time **before parental loss (Pre-bereavement)**, below is a list of possible types of service that could be delivered to families with dependent children. Could you indicate whether or not your organisation provides support by clicking Yes or No in each drop down box.

Q2. And for the **pre-bereavemen**t support you provide, can you tell us who delivers that **support?** ***Please tick*** *all healthcare professionals who deliver each type of service.*

Q3. For families with dependent children, can you tell us to whom you deliver **pre-bereavement** support? *Please tick all that receive each type of service.*

Q4. Still thinking about **pre-** bereavement support, can you tell us where you deliver support? *Please select all boxes that apply.*

Q5. Now, thinking separately about the time **after parental loss** (Bereavement), below is the same list of possible types of support service that could be delivered to surviving partners with dependent children. Could you indicate whether your organisation provides support by clicking Yes or No in the dropdown box.

Q6. And for the **bereavemen**t support you provide, can you tell us who delivers that support? *Please tick all the healthcare professionals who deliver each type of service.*

Q7. For surviving partners with dependent children, can you tell us to whom you deliver **bereavement** support? *Please tick all that receive each type of service.*

Q8. Still thinking about **bereavement** support, can you tell us where you deliver support? *Please select all boxes that apply.*

Q9. Please, could you give us a little more information about the support services you provide for families with dependent children, by describing them in your own words in the box below?

Q10. Could you tell us if you have carried out any evaluations or assessments of the support you provide? If you have, would you be happy to share your findings with us? *Please select one box below.*

- Yes, we have carried out evaluation/assessment and we are happy to share our findings with you.
- Yes, we have carried out evaluation/assessment
- No, we have not evaluated our support provision

Q11. You have indicated that you do not provide specific support within your hospice for families in preparing children for parental death. Is there any alternative help that you refer on to? [No hospice was presented with this question since all provided some support.]

Q12. Please, could you give us some details of services you refer on to? [No hospice was presented with this question since all provided some support.]

Q13. Please tell us in the box below any specific challenges that your hospice has experienced in respect of supporting families with children under 18 years old, either before or after parental loss.
We are particularly interested in challenges that prevent you from providing support, or if you do provide support, challenges that influence delivery.

Section 2

In this second section, we would like to gather a little information about your hospice.

Q14. In which region is your hospice located?

Q15. Thinking about the size of your hospice, how many beds does your hospice have? *Please select the box that applies.*

Q16. Could you please indicate on average, how many new referrals your hospice receives per year. *Please select the box that applies.*

Q17. And finally, we would like to know about the process, if any, your hospice has for gathering information about whether a patient has dependent children. Could you please select the options below that most accurately describes your current procedure. *Please select only one box.*

- We ask at admission and record on a patient's notes.
- We ask at admission, but we do not record on a patient's notes
- We ask informally during a patient's stay and record on their notes
- We ask informally during a patient's stay, but we do not record on their notes
- Our process varies, we have no consistent procedure.
- We do not ask or record at any time.

Q18. You have indicated that your staff ask patients about dependent children. Could you tell us if you provide support to prepare your staff to have these conversations with patients? *Please select Yes/No.*

Q19. Can you describe the support you provide staff to help them have these conversations?

Q20. In this last section, we would like to give you the opportunity to elaborate on any of your answers above. Please feel free to put your comments below. *This question is optional. If you don't need to elaborate, just leave this box blank and click the NEXT button below.*
