# Supplementary material for: The dying parent and dependent children: a nationwide survey of hospice and community palliative care support services
Source: BMJ Support Palliat Care. 2020 Mar 9;12(e5):e696–704. doi: 10.1136/bmjspcare-2019-001947 (PMC9606526; doi:10.1136/bmjspcare-2019-001947)
Supplement: Supplementary data [file bmjspcare-2019-001947supp004.pdf]

The dying parent and dependent children: a nationwide survey of hospice and community palliative care support services.

Supplementary File 4.

Invitation Email

Email heading:

Family Support in Hospices

Dear

I would like to invite you to take part in our scoping survey of UK hospices. We are working with the University of Surrey to identify the best ways hospice healthcare professionals can support patients and their partners to communicate with their children when one parent is dying. As a first stage, we are conducting a short survey of UK hospices to find out what they currently offer.

The survey should take only 10-15 mins to complete, and can be accessed by just clicking this link:

[redacted]

The responses you provide will be completely anonymous. We ask about the support you provide and a few questions about the size and nature of your hospice; if you think another person in the hospice can more easily complete the survey, then please pass on the survey link.

If you have any queries before you begin the survey, please do email me. Alternatively, within the survey you will find contact details for the people at the University of Surrey who are responsible for the study and will be very happy to discuss the survey with you. The survey is being conducted in accordance with the provisions of the University of Surrey's Code on Good Research Practice and University policies

Thank you in advance for your help and contribution.

Best wishes
