# Supplementary material for: The dying parent and dependent children: a nationwide survey of hospice and community palliative care support services
Source: BMJ Support Palliat Care. 2020 Mar 9;12(e5):e696–704. doi: 10.1136/bmjspcare-2019-001947 (PMC9606526; doi:10.1136/bmjspcare-2019-001947)
Supplement: Supplementary data [file bmjspcare-2019-001947supp005.pdf]

The dying parent and dependent children: a nationwide survey of hospice and community palliative care support services.

Supplementary File 5.

Survey Landing Page

Welcome to our survey!

Princess Alice Hospice and the University of Surrey are conducting research to develop ways to enhance the support that is provided to families preparing children for parental death.

Your involvement in this survey is greatly appreciated and your contribution on behalf of your organisation will support our research by helping us build a picture of services currently provided by UK hospices. The survey asks about **services that your organisation provides before and after parental loss for families with children 18 years or under.**

The survey will take 10-15 minutes to complete. By completing the questions, you will be agreeing to the use of the data in meeting the survey objectives. The data you provide will be held in strict confidence in accordance with data protection laws and all information from the questionnaire will be anonymous and not linked to your organisation at any stage. You can complete the survey in more than one session if you require and you can return to the survey as many times as you like until you click the Finish button at the end. If you find that you do not want to complete the survey, just close the survey window and do not return, the data you have entered to that point will be retained and included in the analysis.

If you have any questions about the survey or your participation and would like to talk to somebody, please feel free to get in touch with Jane Cockle-Hearne, who is the Research Fellow administering the survey at the University of Surrey. She can be reached at Tel: [redacted] or Email: [redacted] . If you have any concerns or complaints about any aspect of the study you can contact the Head of Research in the School of Health Sciences at the University, Professor Emma Ream on Tel: [redacted] or Email: [redacted]
