# Supplementary material for: The dying parent and dependent children: a nationwide survey of hospice and community palliative care support services
Source: BMJ Support Palliat Care. 2020 Mar 9;12(e5):e696–704. doi: 10.1136/bmjspcare-2019-001947 (PMC9606526; doi:10.1136/bmjspcare-2019-001947)
Supplement: Supplementary data [file bmjspcare-2019-001947supp006.pdf]

The dying parent and dependent children: a nationwide survey of hospice and community palliative care support services.

Supplementary File 6.

**Type of support delivered by volunteers**	**Pre-Bereavement**		**Bereavement**	
Base: Hospices where volunteers provide support	**n = 57**		**n = 72**	
	n (%)		n (%)	
Written information	**38**	(66.7)	**48**	(66.7)
Signposting to outside agencies, support services	**31**	(54.4)	**38**	(52.8)
Signposting to web-based resources	**29**	(50.9)	**33**	(45.8)
One-to-one support sessions (face-to-face)	**28**	(49.1)	**46**	(63.9)
Groups or pair support sessions	**18**	(31.6)	**35**	(48.6)
One-to-one support sessions (telephone or internet-based)	**17**	(29.8)	**32**	(44.4)
Peer groups meetings	**16**	(28.1)	**34**	(47.2)
CDs/DVDs	**3**	(5.3)	**5**	(6.9)
Other support	**8**	(14.0)	**10**	(13.9)
